# Getting Out of a PCCL: Percutaneous Cholecystolithotomy as a Salvage Treatment Option for Gallstone Removal in Patients Deemed Unfit for Standard Surgical Approaches

**DOI:** 10.1089/cren.2016.0015

**Published:** 2016-02-01

**Authors:** Adam C. Calaway, Michael S. Borofsky, Casey A. Dauw, James E. Lingeman

**Affiliations:** Department of Urology, Indiana University School of Medicine, Indianapolis, Indiana.

## Abstract

Definitive management of acute cholecystitis or symptomatic cholelithiasis in exceedingly high-risk patients remains a clinical dilemma. In certain cases, treatment through a percutaneous approach following standard techniques and principles similar to those of percutaneous nephrolithotomy may be considered. However, one potential challenge, particularly among a high-risk population, is the possible necessity to stay on obligate anticoagulation for pre-existing medical reasons. To date, there have been no prior reports documenting the role of this procedure in patients on systemic anticoagulation, particularly clopidogrel. Here we report a case of a percutaneous cholecystolithotomy performed on an elderly patient unable to stop dual antiplatelet therapy (aspirin and clopidogrel) secondary to recent drug eluting stent placement for myocardial infarction.

## Clinical History

**FIG. 1.**
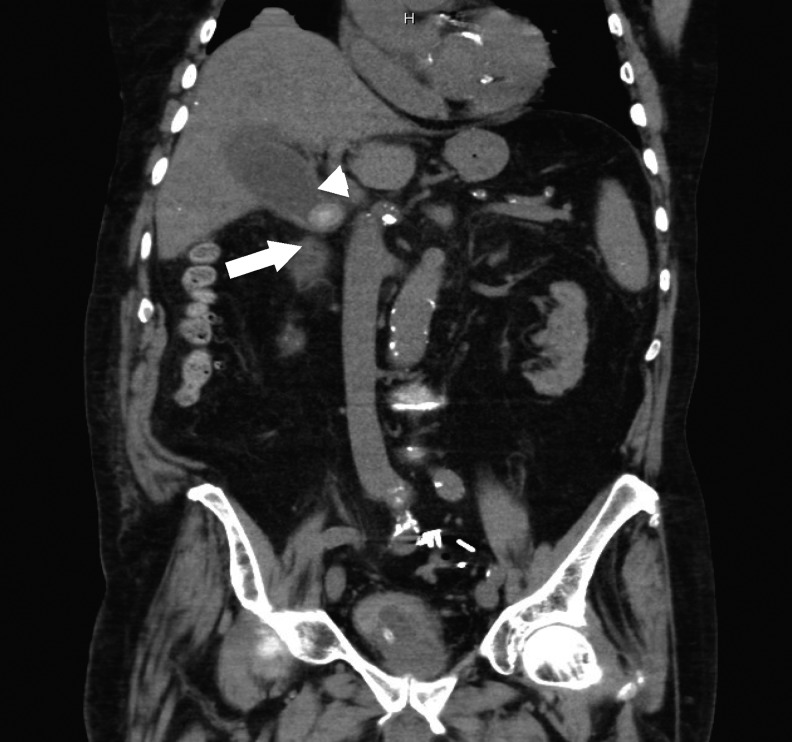
Coronal preoperative CT showing large gallstone obstructing the cystic duct (*arrow*) and pericholecytic fluid (*arrowhead*).

A 91-year-old former physician initially presented to the emergency department with chest pain and was found to have a non-ST elevation myocardial infarction. A cardiac catheterization was performed and three-vessel disease was diagnosed. Three drug eluting stents, adding to the seven pre-existing stents, were placed and the patient was started on aspirin and clopidogrel. Unfortunately, during subsequent hospitalization the patient developed fevers, hypotension, tachycardia, and abdominal pain. An abdominal CT scan was performed that showed a 4 cm gallstone obstructing the cystic duct and significant pericholecystic fluid consistent with acute cholecystitis (Fig. 1).

## Physical Examination

The patient's vitals at the time of his acute episode of cholecystitis were a temperature of 39.2, blood pressure of 75/40, and a heart rate of 115. His abdominal examination showed right upper quadrant tenderness with rebound and a positive Murphy's sign.

## Diagnosis

Laboratory and radiographic studies confirmed the clinical suspicion of acute cholecystitis. His white blood cell count was 18.4. His liver function tests were within normal limits besides his direct bilirubin that was elevated at 0.4 mg/dL. A cholecystostomy tube was placed by interventional radiology and the patient was treated conservatively with prolonged antibiotics directed at the organisms from his biliary cultures (*Escherichia coli and Klebsiella pneumonia*).

## Intervention

**FIG. 2.**
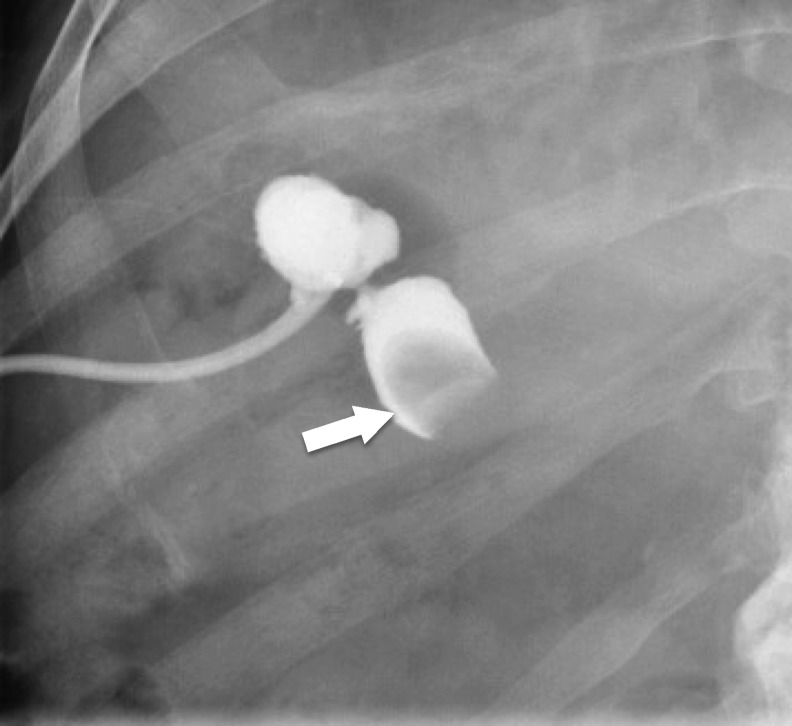
Antegrade cholangiogram showing “hourglass” conformation of the gall bladder with filling defect representing the stone at the neck of the cystic duct (*arrow*) with absent antegrade flow.

The patient was seen and examined by a general surgery team over a 2-month period after diagnosis and the placement of his cardiac stents and cholecystotomy tube. He was deemed to be unfit for definitive cholecystectomy given the obligate necessity to stay on dual antiplatelet therapy as deemed medically necessary by his cardiologist. However, the patient was extremely bothered by his cholecystostomy tube. Antegrade studies through the cholecystotomy tube (Fig. 2) during follow-up showed an unusual hourglass shaped gallbladder. There was no antegrade flow through the cystic or biliary ducts likely secondary to impaction of the stone at the neck of the cystic duct, making tube removal unfeasible. Given his significant discomfort with the tube, he was referred to Urology for consideration of a percutaneous cholecystolithotomy (PCCL) procedure under monitored anesthesia as a minimally invasive approach to treat his stone.

**FIG. 3.**
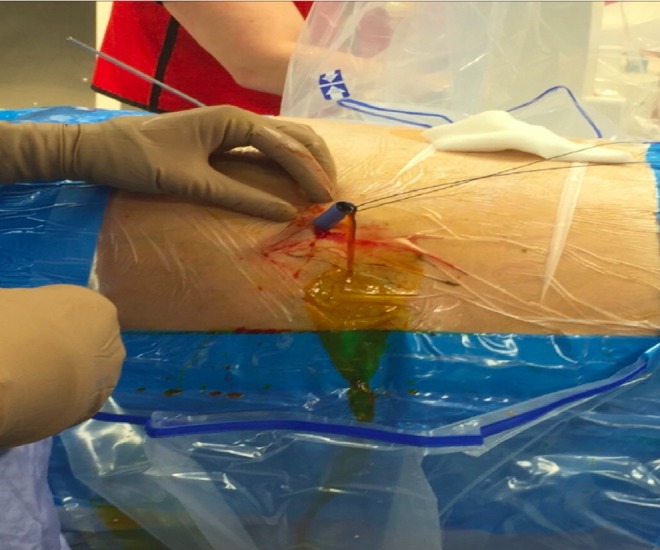
Operative photograph confirming working sheath placement with safety wires in place and bile flowing out of the sheath.

After cardiology clearance, the patient was taken to the operating room for percutaneous removal of his gallstone under monitored anesthesia. The patient was maintained on 81 mg of aspirin and 75 mg of clopidogrel during the perioperative period. He was placed in the supine position with a pad to elevate his right upper abdomen and chest. A Zipwire™ (Boston Scientific Corporation, Natick, MA) was used to cannulate the cholecystotomy tube and this was coiled in the gallbladder. A 5F angiographic catheter was used to exchange the zipwire for an Amplatz Super Stiff™ wire (Boston Scientific Corporation), and a technique for dilation similar to that previously documented for renal caliceal diverticula was used.^1^ A 30F Nephromax™ balloon (Boston Scientific Corporation) was used for initial dilation; however, there was a considerable amount of waist likely secondary to scaring/fibrosis from the prolonged cholecystostomy tube. A 4.5 mm fascial incising needle was employed without success (Cook Medical, Bloomington, IN); therefore, Olympus telescoping metal dilators (Olympus America, Inc., Center Valley, PA) were used to facilitate advancement of the working sheath into the gallbladder. Effective sheath placement into the gallbladder was confirmed with bile flowing from the working sheath (Fig. 3).

Rigid nephroscopy with the 24F Wolf offset nephroscope (Richard Wolf Medical Instruments Corporation, Vernon Hills, IL) was performed. There was minimal bleeding upon entry into the gallbladder. The gallbladder was quite large and the stone was unable to be seen in the cavity of the puncture. Flexible nephroscopy was then performed and a tight opening, between the cavity of entry and the location of the stone previously seen on antegrade studies, was identified. A Zipwire was coiled into the cavity and was later exchanged for a removable core wire for a stiffer working wire. The channel was then localized with the rigid nephroscope and 11F graspers were used to gently dilate into this new cavity. The stone was encountered and easily broken and removed by using an Olympus LUS-2 ultrasonic lithotripter (Olympus America, Inc.).

**FIG. 4.**
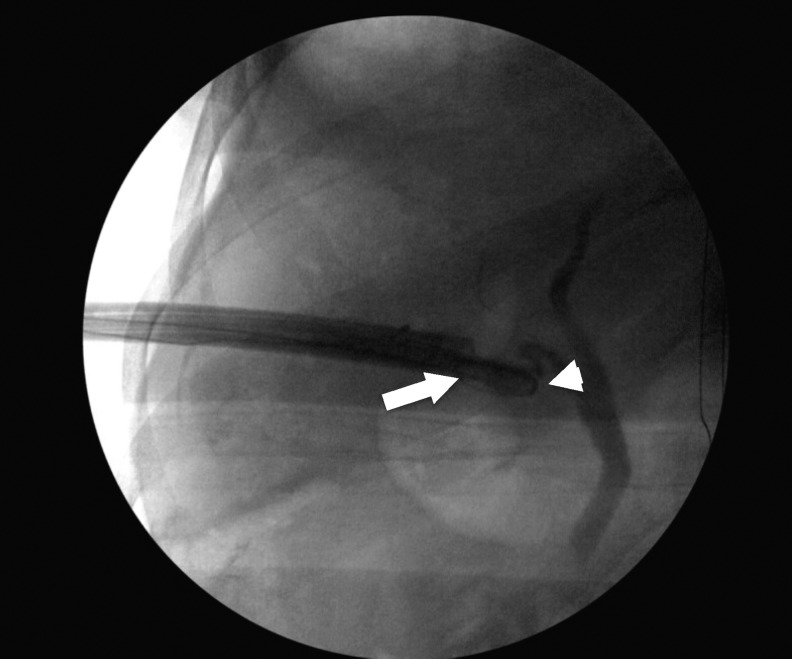
Operative fluoroscopic image after stone removal showing good antegrade flow through the cystic and common biliary duct (*arrow* at end of working sheath and *arrowhead* at tip of foley catheter).

After confirming that all fragments had been removed, a 16F Councill tipped catheter was then left in the gallbladder at the end of the case and stitched into place. Antegrade studies showed opacification of the cystic and common bile duct with drainage into the duodenum (Fig. 4). Estimated blood loss was 30 mL.

## Follow-Up

The patient was managed in the hospital for 2 days postoperatively without issue. His hemoglobin remained stable at 11.6 g/dL, which was similar to his preoperative value of 11.4 g/dL. His cholecysotomy remained in place and had 50 mL of bilious output per day. He was discharged with the tube in place and the decision was made jointly with the general surgery team to leave the cholecystotomy tube for 3 weeks, at which point a repeat antegrade study was normal and the tube was removed without complication or sequelae. The stone analysis showed it was 100% cholesterol.

## Outcomes

PCCL was first described in 1985 as an acceptable operative approach in high-risk patients when laparoscopic or open cholecystectomy was not amendable.^2,3^ However, the exact patient population who are ideal candidates for this approach remains unknown, especially with the relatively minimal morbidity and mortality associated with laparoscopic cholecystectomy in a modern surgical setting. There have been no previous studies documenting PCCL while on systemic anticoagulation. In this case report, we were able to effectively treat a patient through a percutaneous approach for his gallstones while on dual antiplatelet therapy with minimal morbidity and no immediate complications. Notably, blood loss was minimal and did not compromise visualization whatsoever during the procedure despite being on dual antiplatelet therapy.

Recently, a second PCCL was performed on a similarly unhealthy patient with pre-existing end-stage liver disease and coagulopathy associated with liver dysfunction with similar results. This patient was a 55-year-old male with an impacted gallstone and a MELD score of 3.52 and Childs-Pugh score of 8. He had an uncomplicated percutaneous removal of a 1.2 cm impacted stone at the cystic duct. In this case, tract dilation was performed similarly to PCNL as the cholecystotomy tube was recently placed and the tissue was not scared. The stone was easily removed by the ultrasonic lithotripter and rigid graspers. Estimated blood loss was 50 mL. The cholecystotomy tube was removed after an antegrade study showed drainage into the duodenum after 3 weeks.

One of the reported complications of a percutaneous approach is gallstone recurrence. Researchers have postulated that by not removing the gallbladder mucosa, and thus not interrupting the pathogenesis of gallstones, the rates of stone recurrence could be fairly significant. In fact, a study reported that the recurrence rates in 439 patients who underwent PCCL were 41% after 10 years with 21% of these patients ultimately requiring extirpative surgery.^3^ However, colleagues in endourology previously reported a case describing a gallbladder mucosa fulguration technique, similar to the technique used in percutaneous caliceal diverticula management, to induce coagulation necrosis of the gallbladder wall to limit its secretory function. In the solitary patient treated with this technique, Andonian et al. reported an absence of recurrence after 1 year of clinical follow-up.^4^ Although a technique with promising utility to minimize recurrence given the success in similar urologic operations, fulguration was not undertaken in this patient given the theoretical concerns for gallbladder perforation and the continued anticoagulation. Nevertheless, we believe that the PCCL operation is safe and feasible in patients on systemic anticoagulation who are not candidates for cholecystectomy and certainly worth considering if facile with percutaneous endourologic techniques.

## Abbreviations Used

CT: computed tomography
PCCL: percutaneous cholecystolithotomy
PCNL: percutaneous nephrolithotomy
